# Exploring the acceptability of HIV partner notification in prisons: Findings from a survey of incarcerated people living with HIV in Indonesia

**DOI:** 10.1371/journal.pone.0234697

**Published:** 2020-06-30

**Authors:** Gabriel J. Culbert, Agung Waluyo, Valerie A. Earnshaw

**Affiliations:** 1 Department of Health Systems Science, College of Nursing, University of Illinois at Chicago, Chicago, IL, United States of America; 2 Center for HIV/AIDS Nursing Research, Faculty of Nursing, Universitas Indonesia, Depok, Indonesia; 3 Department of Human Development and Family Sciences, University of Delaware, Newark, DE, United States of America; NPMS-HHC CIC / LSH&TM, UNITED KINGDOM

## Abstract

Assisted HIV partner notification services provide a safe and effective way for people living with HIV (PLHIV) to inform their partners about the possibility of exposure and to offer them testing, treatment, and support. This study examined whether or not PLHIV in prison might be willing to participate in assisted HIV partner notification services and their reasons for and against disclosing their HIV-positive status to their partners. PLHIV (n = 150) recruited from Jakarta’s two largest all-male prisons completed an interviewer-administered questionnaire collecting demographic and risk behavior data, and attitudes toward HIV disclosure and partner services. Among those who were sexually active and/or injecting drugs before incarceration, two-thirds (66.4%, 91/137) endorsed provider referral as an acceptable way to notify their sex partners, and nearly three quarters (72.4%, 89/123) endorsed provider referral to notify their drug-injecting partners. Only a quarter (25.1%) of participants reported that their main sex partner had ever received an HIV test. Participants with anticipated stigma were less likely to endorse provider referral for sex partners (adjusted odds ratio [aOR] = 0.58, 95% CI: 0.35, 0.96) and drug-injecting partners (aOR = 0.54, 95% CI: 0.29, 1.00). Relationship closeness was associated with higher odds of endorsing provider referral for drug-injecting partners (aOR = 2.08, 95% CI: 1.25, 3.45). Protecting partners from infection and a moral duty to inform were main reasons to disclose, while stigma and privacy concerns were main reasons not to disclose. Most incarcerated PLHIV have at-risk partners in the community who they would be willing to notify if provided with assistance. Assisted partner notification for prison populations offers a promising public health approach to accelerate diagnosis, treatment, and prevention of HIV infection in the community, particularly among women.

## Introduction

HIV testing is a major gap in the treatment cascade worldwide and the main obstacle to treatment in many low- to middle-income countries (LMICs) [1], including Indonesia where in 2019 only half (51%) of the estimated 640,000 people living with HIV (PLHIV) were diagnosed [2]. To increase diagnosis, HIV testing should target populations with the highest HIV incidence. Two important populations for HIV testing are people in prison, who are disproportionately affected by HIV [3], and their sex and drug-injecting partners in the community, who also are at risk [4–6]. An estimated 389,000 PLHIV are incarcerated worldwide at any given time and many more transition through jails and prisons each year [3]. Although many PLHIV are diagnosed in prison, their sex and drug-injecting partners in the community may be missed for HIV testing and treatment [4].

Assisted HIV partner notification services refers to when consenting PLHIV are assisted by a trained healthcare provider to disclose their HIV status or to anonymously notify their sex and/or drug-injecting partners of their potential exposure to HIV infection [7]. Although many PLHIV disclose their HIV status to partners without assistance [8, 9], provider-assisted approaches consistently increase notification and testing of partners compared to approaches that rely only on patients telling their partners [10–14]. On the basis of systematic reviews [11, 15, 16] and recent clinical trials [12, 14], the World Health Organization recommends that healthcare providers encourage and, if asked, actively assist PLHIV to notify their partners and give them options for HIV testing [10]. This policy recommendation, however, includes no evidence from criminal justice settings, which is an important gap in the scientific literature given the important role of prisons in HIV prevention globally, and the likely challenges to engaging prisoners in partner notification.

Available evidence suggests that PLHIV are mostly willing to disclose their HIV status and to participate in provider-assisted HIV partner notification [8, 9, 17]. Yet, prisons are distinct psychosocial settings in which disclosure of one’s HIV status may be more stigmatizing [18–20]. Disclosure decisions are primarily influenced by a person’s *approach* and *avoidance goals*, which refer to the perceived positive and negative consequences of disclosure [21, 22]. *Approach goals* are perceived positive outcomes, such as the desire for a closer relationship, that can motivate PLHIV to disclose their HIV status to others [21–24]. *Avoidance goals* are perceived negative outcomes, such as stigma, that can cause PLHIV to avoid disclosing their HIV status to others. Individual decisions about whether and how to disclose one’s HIV status also may be influenced by a person’s past experiences with HIV disclosure [21, 22] and relationship characteristics such as the desire for children and/or greater intimacy with a partner [25, 26]. As researchers begin to examine the possibilities for HIV partner notification in new settings and populations, it is worth examining how PLHIV weigh these psychosocial and interpersonal factors in reaching a decision to notify their partners and choose among the various referral methods.

Indonesia, the world’s fourth most populous country, provides an important context in which to explore the potential benefits of offering HIV partner services in prisons. Indonesia was one of the first Asian countries to develop a national HIV/AIDS strategy to prevent, diagnose, and treat HIV in prisons [27]. Most of Indonesia’s ~260,000 prisoners are male [28] and 6%-8% are people who inject drugs (PWID) [29]. HIV prevalence in male prisoners is 1.1% [30], but higher in specialized narcotic prisons (6.5%-7.2%) where many PWID are incarcerated [31]. Implementing the National Strategy has brought progress and challenges. Under Indonesian national guidelines, antiretroviral therapy (ART) is recommended for all PLHIV, including those in prison, regardless of CD4^+^ cell count [32]. Recent evidence suggests that a substantial proportion of PLHIV in prison are individuals who were first diagnosed and initiated treatment within prison [33]. Nevertheless, HIV stigma and lack of information about the benefits of ART may contribute to low levels of ART adherence in prisons [18, 33], and mortality remains high among PLHIV within prison and after release [34]. Likewise, harm reduction initiatives in prisons, including condom and needle-syringe distribution, and methadone treatment for opioid use disorder have achieved only modest diffusion [35], despite ample evidence of HIV risk behaviors in prison [36, 37].

In the Indonesian community, HIV transmission is increasingly from key populations to their sex partners [38, 39]. One third of the estimated 46,000 annual new infections in 2018 were among women [2] whose main risk factor for infection is having a male partner who injects drugs or buys sex [40]. HIV testing remains low due to low risk perception and high levels of HIV stigma [41–45]. Consequently, many PLHIV (34%-47%) are diagnosed at an advanced stage of infection [46, 47], and women are often diagnosed only after they develop symptoms or lose a partner to HIV infection. Although active contact tracing results in earlier diagnosis [11], many countries, including Indonesia, lack specific policies on HIV partner notification [48]. Guidelines for HIV testing in Indonesia recommend testing spouses of PLHIV, but do not specify how or who should provide this service [48], and studies indicate low awareness of partner notification in key populations [49]. Given the high rates of new HIV diagnosis in prisons, the lack of partner services in these settings is a conspicuous missed opportunity.

Recommendations for developing behavioral interventions often include a stage of basic social sciences research that occurs prior to testing the intervention [50]. In this study, we examined 1) the acceptability of anonymous notification by a health service provider (i.e. provider referral) and 2) *approach* and *avoidance* goals HIV status self-disclosure (i.e. passive referral), which is a key component of other provider-enabled approaches, including dual referral and contract referral) [51]. Drawing on prior theoretical work [52], we defined acceptability as the extent to which PLHIV endorsed provider referral as an appropriate or potentially useful approach for notifying people with whom they had shared an exposure to the virus. Results offer insights as to the future viability of partner services in the prison context and have implications for developing partner services in Indonesia and other countries where HIV prevalence is high in prisons [53, 54].

## Methods

### Study design

This cross-sectional study utilizes data collected from participants enrolled in a randomized controlled trial of the Adherence Through Home Education and Nursing Assessment (ATHENA) intervention (NCT03397576) [55]. Briefly, incarcerated persons who were Indonesian citizens ≥18 years of age and HIV diagnosed (self-reported and confirmed with rapid HIV antibody test at enrollment) were randomized to receive the adapted ATHENA intervention [56] or treatment as usual. Participants randomized to ATHENA received medication adherence counseling within prison followed by home visits after prison release to reinforce adherence behaviors. Treatment as usual included screening and prophylaxis for opportunistic infections, treatment with ART, and adherence monitoring. Analyses presented here utilize data collected from participants during their initial study visit in prison.

### Study setting

Participants were recruited from February 2017 to March 2018 from two male prison facilities in Jakarta, Indonesia, one of which was a specialized narcotic prison that houses persons charged with drug-related offenses. Both facilities are extremely overcrowded and HIV prevalence rates are higher compared to prisons in other parts of the country [57]. HIV testing is offered to all persons during intake into jails and also at the request of inmates [58]. Persons diagnosed with HIV may access ART at no cost through prison-based HIV subspecialty clinics located within each prison.

### Recruitment and data collection

The study was introduced to HIV-diagnosed patients during their regularly scheduled clinic visits in prison. Researchers conducted informed consent procedures in private rooms away from other prisoners, correctional officers, and prison medical staff. To minimize missing data, questions were administered by trained research assistants using a secure web-based application. Participants were actively monitored during study procedures to ensure that they adequately understood questions and response choices. To reduce social desirability bias, research assistants took time to establish trust and build rapport before asking potentially sensitive questions [59]. All questionnaires and instruments underwent a rigorous forward-backward translation process and pre-testing before the study.

### Study measures

The two main outcomes in this study were: 1) the acceptability of provider referral as a method for notifying sex and drug-injecting partners, and 2) *approach* and *avoidance goals* for HIV status self-disclosure. To assess the acceptability of provider referral, researchers first read a script describing provider referral as a process in which a nurse or doctor helps to confidentially notify and offer HIV testing to partners who may have been exposed to HIV through sex or needle-sharing. Participants then were asked whether or not they would be willing to let a nurse or doctor confidentially notify their 1) sex and/or 2) drug-injecting partner(s) in the community of possible HIV exposure, with response options for *yes* and *no*. Researchers explained that partner notification questions were only to ascertain whether such services were of interest to participants and that researchers had no plans to collect information about or to notify partners.

Researchers assessed participants’ *approach* and *avoidance goals* for HIV status self-disclosure using statements from The Reasons for Disclosure Questionnaire, which measures reasons for disclosure/non-disclosure after an HIV diagnosis [60]. The Reasons for Disclosure Questionnaire consists of 24 statements measuring 5 reasons for disclosing (catharsis, duty to inform, test the other persons’ reactions, relationship support and similarity), and 6 reasons for non-disclosure (privacy, self-blame, communication difficulties, fear of rejection, protecting the other, and superficial relationship). Participants were asked which of the various reasons might be most influential in deciding whether or not to disclose their HIV status to 1) sex and/or 2) drug-injecting partners with whom they may have shared an HIV exposure.

Demographic characteristics included age, education, marital status, and length of incarceration. HIV treatment factors included diagnosis date and location (i.e., prison or community), current or previous use of ART, and CD4^+^ T-lymphocyte count. Frequency of drug use/injection in the three months before incarceration was assessed using the Texas Christian University Drug Screen, which was previously adapted [37, 61]. To assess pre-incarceration sex behaviors, participants were asked whether or not they were sexually active (defined as penile-vaginal or penile-anal intercourse) with men, women, and/or transgender women (known locally as *waria*) in the 6 months before the current prison term. To reduce social desirability bias, participants were first asked whether they preferred sex with men, women, *waria* or some combination of the three. To reduce recall bias, questions about pre-incarceration drug and sex risk behaviors used an “anchoring event”. Because this study sought to ascertain the acceptability of provider referral for notifying partners in the community, participants were not asked about sexual activity in prison.

HIV stigma was assessed using a multidimensional measure of HIV stigma previously adapted for the Indonesian prison setting [18], with subscales measuring anticipated stigma, internalized stigma, and intersectional stigma (i.e., stigma associated with substance use and incarceration). Participants indicated the frequency of stigma experiences on a 5-point Likert-type scale from *never* to *always*, with higher scores indicating higher perceived stigma. Reliability was high (15 items; α = 0.91). Disclosure history was assessed using a multiple-response question asking participants whether they had disclosed their HIV status to a close friend, family member, sex partner, or drug-injecting partner. Participants who had not disclosed their status to anyone were classified as having never disclosed.

Three items adapted from the Unidimensional Relationship Closeness Scale [62] were used to assess how close participants were to their sex and drug-injecting partner(s). Items included, *How close are you to your sex (drug-injecting) partners*, *How important are your sex (drug-injecting) partners*, and *How often do you see your sex (drug-injecting) partners*. Responses were given on a four-point Likert-type scale. Preliminary analyses suggested that the items were reliable (sex partners, α = 0.82; drug-injecting partners, α = 0.74). Composite scores were created to indicate relationship closeness, with higher scores indicating greater closeness.

The multidimensional health locus of control scale was used to assesses the degree to which participants attributed health outcomes to their own actions (internal subscale), fate or supernatural influences (chance subscale), or the actions of family members and physicians (powerful others subscale) [63]. Orthogonal subscales consisted of 6 Likert-type items to which respondents rated their level of agreement from *strongly agree* to *strongly disagree*. Items were reworded to focus specifically on HIV-related health problems. Cronbach’s alphas for the internal, chance, and powerful others subscales were .59, .80, and .55, respectively.

### Data analysis

This study was a secondary data analysis of pilot data to generate hypotheses for a future study. Descriptive statistics were used to characterize the sample and examine pre-incarceration HIV treatment and behavioral risk factors. Due to low missing data, no imputation was required. We conducted separate bivariate and multivariate analyses to examine associations with willingness to endorse provider referral to notify 1) sex partners, limiting our analysis to participants who were sexually active in the 6 months before incarceration; and 2) drug-injecting partners, limiting our analysis to participants with a pre-incarceration drug injection history. To fit a final model with few parameters, we selected for inclusion in our model socio-demographic variables with a significant bivariate association (*p*<0.05; i.e., age, diagnosis in prison or community) or theorized to influence acceptability of provider referral (i.e., stigma, disclosure history, relationship closeness, health locus of control). Finally, we examined participants’ *approach* and *avoidance goals* for HIV status self-disclosure to sex/drug-injecting partners using frequencies and pie charts.

### Ethics statement

All procedures were conducted in accordance with international guidelines for research with prisoners [64]. Study protocols were reviewed by institutional review boards at the University of Illinois at Chicago and *Universitas Indonesia*, with additional oversight from the U.S. Office of Human Research Protections. Subjects were selected equitably and without the involvement of prison staff. All subjects provided written informed consent, specifying that research participation was voluntary and would result neither in benefit nor punishment.

## Results

### Participant characteristics

Researchers screened 164 persons for eligibility, of whom 150 met inclusion criteria and completed baseline data collection. Participant characteristics are shown in Table 1. Participants were 34.3 years of age on average and about half had completed high school. Participants were HIV-diagnosed for an average of 4.7 years and almost half were HIV diagnosed during the current prison term. Most participants reported a history of drug injection and many injected drugs immediately before incarceration.

**Table 1:** Characteristics of HIV-infected males in prison (n = 150).

Variable	n	(%)
**Demographic**		
Age in years (mean ± SD)	34.3 ± 7.5	
Finished high school	85	56.7
Married/in a relationship	84	56.0
Incarcerated in a narcotic prison	112	74.7
Years incarcerated (mean ± SD)	2.0 ± 1.3	
**HIV treatment**		
Years since HIV diagnosis (mean ± SD)	4.6 ± 4.1	
HIV diagnosed during current prison term	64	42.7
Receiving ART before incarceration	41	27.3
CD4^+^ T-lymphocyte cells/μL (mean ± SD)	289 ± 179	
Currently utilizing ART	110	73.3
Self-reported “perfect” ART adherence (n = 110)	28	25.5
**Substance use**		
Any history of drug injection	123	82.0
Pre-incarceration substance use ^a^	146	97.3
Pre-incarceration drug injection ^a^	66	44.0
Within-prison substance use ^b^	93	62.0
Within-prison drug injection ^b^	14	9.3

ART, antiretroviral therapy; SD, standard deviation.

^a^ three months before incarceration; includes only opioids, stimulants, and sedatives.

^b^ during the current prison term; includes only opioids, stimulants, and sedatives.

### Pre-incarceration HIV risk behaviors

Most participants (91.3%, 137/150) were sexually active in the six months before incarceration (Table 2). Participants identified a total of 469 sex partners in the six months before incarceration, including women (75.2%, 353/469), men (24.0%, 113/469) and male-to-female transgender persons (<1.0%, 3/469). Nearly half of those who were sexually active before incarceration (48.1%, 66/137) reported sex with just one person, while half (51.8%, 71/137) reported sex with two or more partners. Most of those who were sexually active before incarceration acknowledged having a main sex partner (someone with whom they had a regular and committed sexual relationship), and about half acknowledged having casual sex partners during that time. Condom use with sex partners was low overall (between *seldom* and *sometimes*), and participants were significantly less likely to have used condoms with main partners compared to non-main partners (*p* = 0.001). A few participants also reported injecting drugs or sharing needles with main and casual sex partners before incarceration. Only a quarter of participants reported that their main sex partner had ever received an HIV test.

**Table 2:** Pre-incarceration HIV risk behaviors among HIV-infected male prisoners (n = 150).

Variable	n	(%)
**Pre-incarceration sex risk behaviors**		
Sexually active ^a^	137	91.3
Sex with female partners only (n = 137)	135	98.5
Median number of sex partners (mode, range, total)	2 (1, 1–56, 469)	
***Main partner*** (n = 135)		
Always used condoms	12	8.8
Injecting drugs or sharing needles	10	7.4
Ever tested for HIV	34	25.1
***Casual partner(s)*** (n = 71)		
Always used condoms	10	14.0
Injecting drugs or sharing needles	5	7.0

^a^ engaged in penile-vaginal/anal intercourse in the six months before incarceration.

### Psychosocial factors and willingness to endorse provider referral

Table 3 shows participant scores on psychosocial factors theorized to influence willingness to endorse provider referral. On average, participants experienced moderate levels of anticipated stigma (mean = 2.7, SD = 1.1), internalized stigma (mean = 2.6, SD = 1.3) and intersectional stigma (mean = 2.8, SD = 1.1). Health locus of control was highest for the internal subscale. Participants reported greater relationship closeness to sex partners (mean = 3.15, SD = 0.94) compared to drug-injecting partners, (mean = 2.61, SD = 0.97). Although most participants had disclosed their HIV status to a close friend or family member, fewer had disclosed their HIV status to sex or drug-injecting partners.

**Table 3:** Psychosocial factors and willingness to endorse provider referral (n = 150).

Variable	Mean	± SD
**HIV stigma** (range: 1–5)		
Anticipated stigma	2.72	1.16
Internalized stigma	2.65	1.38
Intersectional stigma	2.86	1.12
**Relationship closeness** (range: 1–4)		
Sex partner(s)	3.15	0.94
Needle-sharing partner(s)	2.61	0.97
**Health locus of control** (range: 1–5)		
Internal	3.98	0.43
Chance	3.34	0.70
Powerful others	3.70	0.47
**HIV**^**+**^ **status disclosure**	**n**	**%**
Has disclosed to friend or family member	132	88.0
Has disclosed to ≥ 1 sex partner ^a^	41	27.3
Has disclosed to ≥ 1 drug-injecting partner ^b^	12	9.8
**Willingness to endorse provider referral to notify partners**		
Sex partners ^a^	91	66.4
Drug-injecting partners ^b^	89	72.4

SD, standard deviation.

^a^ among participants reporting sexual activity in the six months before incarceration (n = 137).

^b^ among participants reporting a history of drug injection (n = 123).

Two-thirds of participants who were sexually active immediately before incarceration (66.4%, 91/137) responded that they would be willing to let a nurse or doctor notify their sex partner(s) in the community of possible HIV exposure. Among participants with a history of drug injection, nearly three quarters (72.4%, 89/123) endorsed provider referral as an acceptable method to notify their drug-injecting partners in the community.

### Associations with willingness to endorse provider referral

Multivariate associations with willingness to endorse provider referral from the logistic regression are shown in Table 4. In an adjusted model, each one unit increase in anticipated stigma was associated with a 42% lower odds of being willing to endorse provider referral to notify sex partners (adjusted odds ratio [aOR] = 0.58, 95% CI: 0.35, 0.96), and a 46% lower odds of endorsing provider referral to notify drug-injecting partners (aOR = 0.54, 95% CI: 0.29, 1.00). Conversely, relationship closeness was associated with a higher adjusted odds of endorsing provider referral to notify drug-injecting partners (aOR = 2.08, 95% CI: 1.25, 3.45). The adjusted odds of endorsing provider referral to notify sex partners decreased with age and was lower for those diagnosed during the current prison term, those who had not yet disclosed their HIV status to friends or family members, and those with higher chance locus of control scores; however, these associations were not statistically significant.

**Table 4:** Associations with willingness to endorse provider referral to notify sex and drug-injecting partners from multivariate logistic regression.

	Sex partners ^a^		Drug-injecting partners ^b^	
Variable	aOR ^c^	95% CI	aOR ^c^	95% CI
Age	0.94^†^	0.89, 1.01	1.05	0.97, 1.13
Diagnosed in prison ^d^	0.48^†^	0.21, 1.11	0.51	0.19, 1.34
Stigma				
Anticipated	0.58*	0.35, 0.96	0.54*	0.29, 1.00
Internalized	1.37	0.90, 2.08	1.44	0.87, 2.40
Intersectional	1.22	0.82, 1.83	1.11	0.67, 1.84
Has not disclosed HIV status ^e^	0.33^†^	0.10, 1.16	0.48	0.10, 2.25
Relationship closeness	1.12	0.73, 1.71	2.08**	1.25, 3.45
Health locus of control				
Internal	1.88	0.61, 5.77	2.25	0.65, 7.83
Chance	0.55^†^	0.28, 1.07	0.64	0.32, 1.31
Powerful others	0.96	0.35, 2.62	2.35	0.74, 7.42

aOR, adjusted odds ratio; CI, confidence interval.

^†^ p<0.10

*p<0.05

**p<0.01.

^a^ analysis limited to participants reporting sexual activity in the three months before incarceration (n = 137).

^b^ analysis limited to participants reporting a history of drug injection (n = 123).

^c^ adjusted for all other co-variates in the model.

^d^ compared to participants diagnosed before the current prison term.

^e^ compared to participants who have disclosed their HIV+ status to ≥ 1 friend or family member.

### Approach and avoidance goals for HIV status disclosure

**Fig 1:**
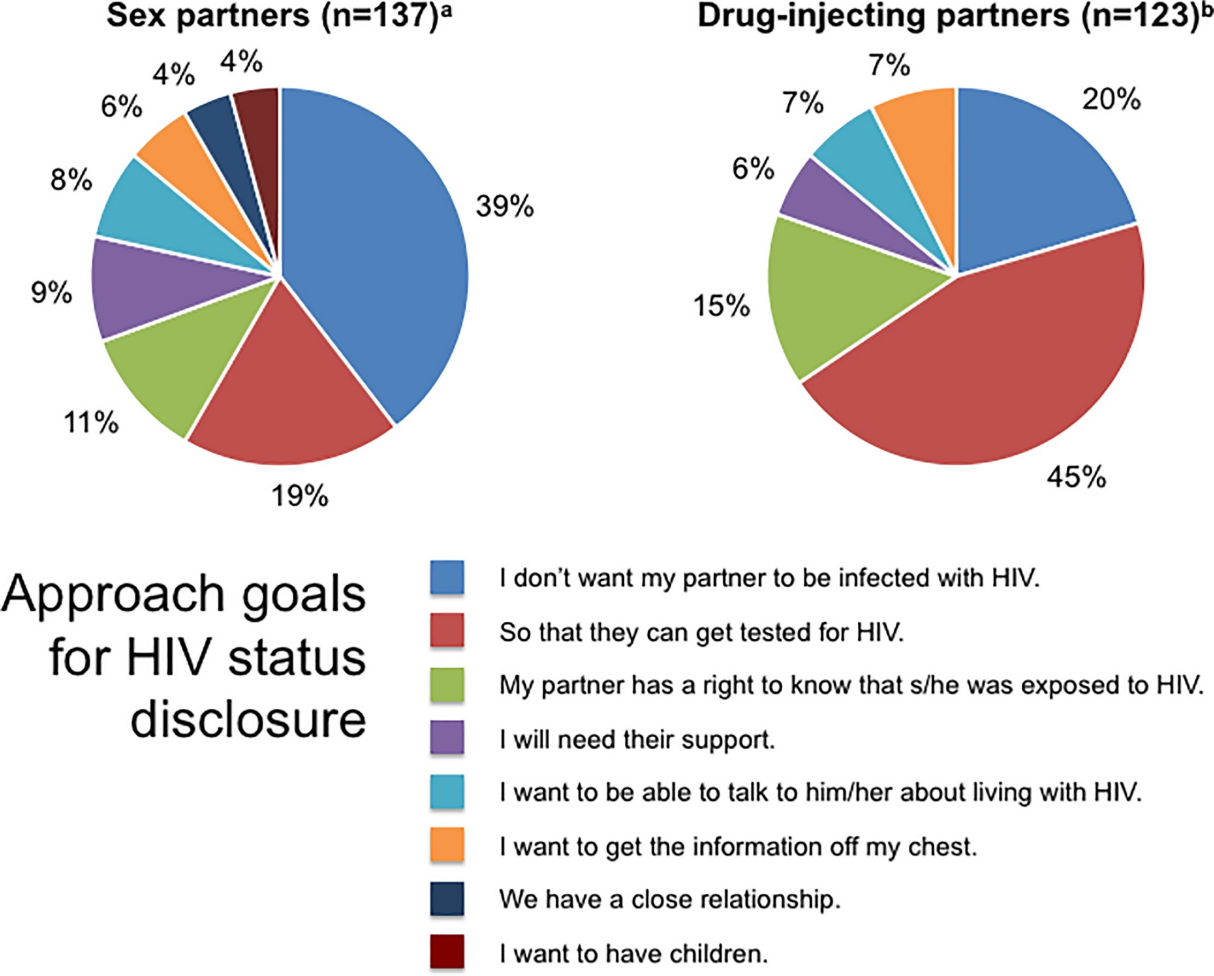
Approach goals for HIV status disclosure. ^a^ n = number of participants who were sexually active in the 6 months before incarceration. ^b^ n = number of participants who injected drugs before incarceration.

Fig 1 shows *approach goals* for HIV status self-disclosure. The reasons most frequently given by participants for wanting to disclose their HIV status to their sex partners (i.e. approach goals) were to prevent partners from becoming infected, to allow partners to be tested for HIV, and to inform partners about the possibility of HIV exposure. Participants’ main reasons for wanting to disclose their HIV status to drug-injecting partners were to allow those partners to be tested for HIV, to prevent partners from being infected, and to inform partners about the possibility of HIV exposure. Participants also indicated that the desire for social support and wanting to reveal information that had caused them emotional discomfort were important reasons for disclosing their HIV status to sex and drug-injecting partners.

**Fig 2:**
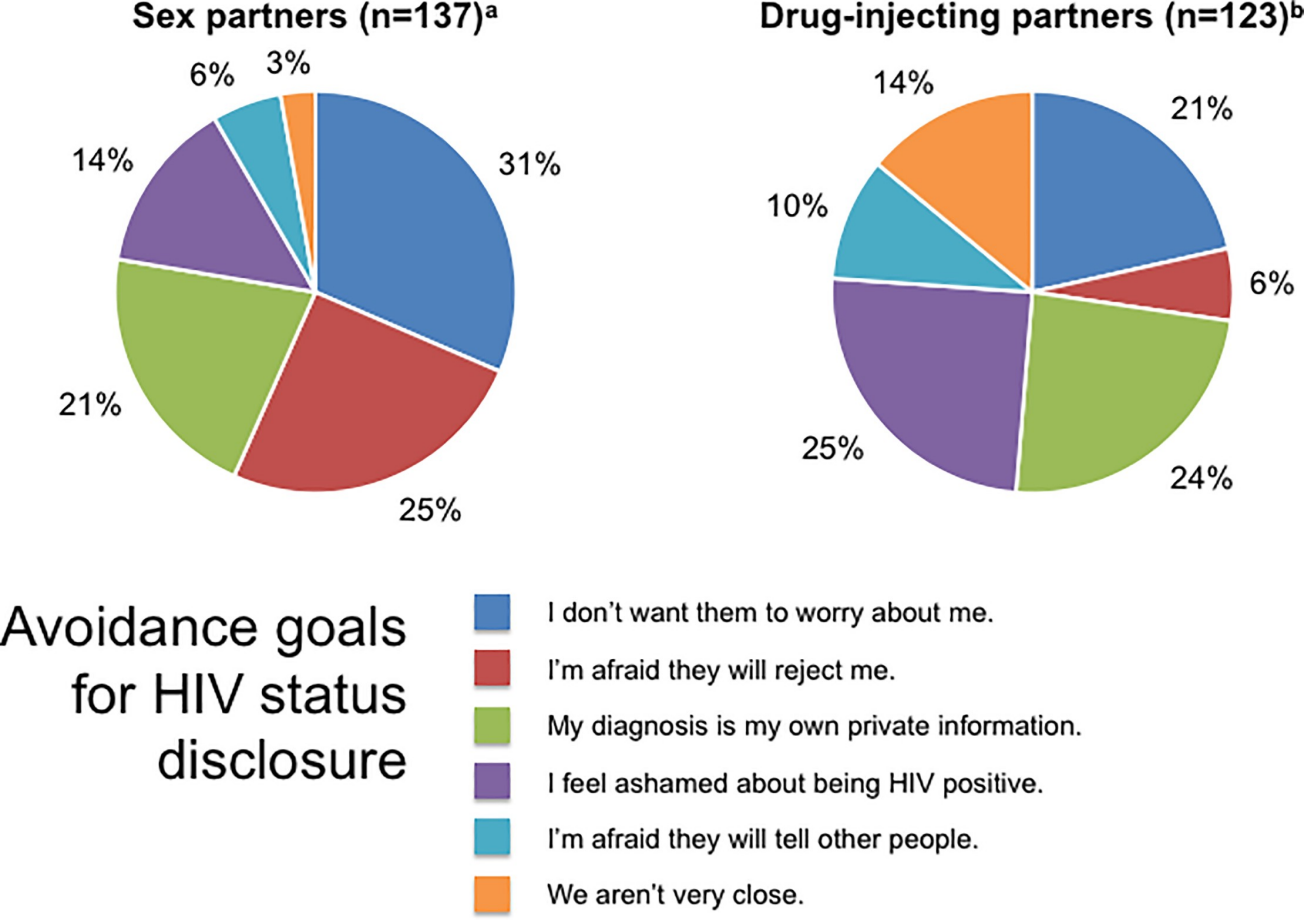
Avoidance goals for HIV status disclosure. ^a^ n = number of participants who were sexually active in the 6 months before incarceration. ^b^ n = number of participants who injected drugs before incarceration.

Fig 2 shows *avoidance goals* for HIV status self-disclosure. The main reasons given by participants for not wanting to disclose their HIV status to sex partners (i.e. avoidance goals) were not wanting to burden partners with that information, fear of rejection, loss of privacy, and internalized stigma. Main avoidance goals for disclosure to drug-injecting partners were internalized stigma, loss of privacy, not wanting to burden partners with that information, lack of relationship closeness, loss of confidentiality, and fear of rejection.

## Discussion

In this study, researchers examined the acceptability of anonymous notification by a health service provider (i.e. provider referral) and approach and avoidance goals for HIV status self-disclosure in a sample of incarcerated PLHIV, most of whom were sexually active and using drugs before incarceration. We found that two-thirds (66.4%) of PLHIV endorsed provider referral as an acceptable method to notify their sex partners and nearly three quarters (72.4%) endorsed provider referral to notify their drug-injecting partners. Although participants were presented only with a hypothetical scenario (i.e., participants were told that researchers had no intention to notify partners), these high levels of acceptance held constant regardless of the index patient’s age, sexual orientation, or past disclosure experiences, signaling an important opportunity to implement WHO-recommended HIV partner services in prisons. Nevertheless, our findings also show that anticipated stigma, fear of rejection, and privacy concerns may be important barriers to assisted partner notification and that researchers and service providers will need to consider carefully how best to address these concerns to promote the safety of PLWH and ensure the broadest possible participation. Because people in prison may be estranged from or have difficulty contacting their partners, the service option of provider referral offers a practical alternative that relieves the burden of informing a partner, offers anonymity, and expedites notification.

A main finding from this study was that sex partners of incarcerated PLHIV represent a sizable at-risk population, composed primarily of women, who may be missed for HIV testing and/or treatment. This finding is consistent with previous research describing the neglected health needs of women with an incarcerated male partner [4, 6], and of particular relevance in Indonesia, where heterosexual HIV transmission is increasing and women are typically diagnosed only because of a husband’s illness [39]. Our sample was comprised entirely of HIV-positive and presumptively cis-gender males, most of whom were sexually active, not receiving treatment with ART, and reported low rates of consistent condom use before incarceration–a time when most were likely already infected with the virus. Nearly half of PLHIV in this sample were diagnosed during the current prison term, yet few had disclosed their HIV status to a sex partner since their diagnosis or indicated that their sex partner had ever received an HIV test. PLHIV in this study identified a total of 469 sex partners in the 6 months before incarceration, including men, women and transgender persons. Although these findings signal an important opportunity to provide partner services, further studies are needed to ascertain the characteristics of partners whom incarcerated PLHIV actually choose to notify, the ability of index patients to provide contact information for their partners, and whether or not PLHIV trust that healthcare providers will maintain strict confidentiality and provide supportive services to their partners. Nevertheless, findings here support the inference that assisted partner services could, in theory, lead to more of these partners being tested, learning their HIV status, accessing treatment in the community, and taking steps to reduce their risk of future exposure.

Findings from this study indicate ongoing HIV transmission risk to index patients and their partners and suggest that partner notification could play an important role in mitigating the risk of future exposure. For example, some PLHIV continued to inject drugs and share injecting equipment within prison. These activities, coupled with low rates of ART adherence within prison, pose a major challenge for preventing the emergence and transmission of drug-resistant HIV. Recent research in Indonesia demonstrates that incarceration is the single-most important risk factor for acquiring drug resistant HIV in PWID [65]. At present, interventions for PLHIV in prison focus mainly on behavioral risk reduction and increasing utilization of ART and opioid agonist treatment for the treatment of opioid use disorder [66]. Although indispensable for improving health outcomes and reducing HIV transmission, these interventions require high levels of adherence in order to be effective and do not address the needs of partners who may already have shared an exposure. Contact tracing for key populations and their sex partners, who together account for 47% of new infections globally [67], also is needed to reduce the spread of HIV in prisons and beyond [68].

A key finding from this study was that participants with higher anticipated stigma were less likely to endorse provider referral to notify sex or drug injecting partners. Previous research suggests that PLHIV often experience anxiety about disclosing their HIV status to others [69] and that those with higher anticipated stigma and fewer coping resources are less likely to disclose their HIV status or agree to notify partners [70]. Although HIV partner notification rarely results in social or economic harms for people in community settings [71, 72], the risks for people in prison are unknown. PLHIV in prison suffer the social costs of multiple stigmatizing identities [18, 73], yet have fewer resources for coping with stigma. Disclosure that results in loss of social support may have greater negative consequences for PLHIV who often depend on their partners for housing, financial security, and treatment support after prison release. Yet, it is also possible that partner notification could lead to greater relationship closeness and stability [74], which is an especially important outcome in the context of prisons given that incarceration often destabilizes the lives of inmates and their sex partners in ways that increase their risk of HIV exposure [75, 76]. We have argued elsewhere that implementing HIV partner services in prisons raises serious ethical challenges [77], including questions about the ability of service providers within these settings to ensure that partner services are provided consistent with rights-based WHO guidelines [78]. Interventions that give PLHIV in prison greater control over their health information and health care decisions are likely to reduce stigma and improve acceptance and utilization of partner services.

Findings here suggest that a substantial proportion of incarcerated PLHIV (72.4%) also may be open to notifying individuals with whom they shared a drug-related exposure before incarceration. This finding has particular significance in Indonesia and other countries where a majority of those in prison are PWID, and drug injection that occurs within prison and after prison release continues to be an important source of new infections [79]. PLHIV in this study were more likely to endorse provider referral to notify drug-injecting partners if they perceived those relationships as close, important, and characterized by frequent interaction. This finding is consistent with previous work suggesting that people are more likely to disclose in close relationships and that disclosure leads to relationship closeness [23–26]. In this study, few PLHIV with a history of drug injection had disclosed their HIV diagnosis to a drug-injecting partner, perhaps because incarceration made it difficult to contact these partners. Yet, most indicated an interest in disclosing their HIV status to drug-injecting partners in order to allow those partners to receive HIV testing and prevention services. Although few studies have examined partner notification for PWID, a study of outreach-assisted partner notification in the U.S. found that a majority of PWID requested assistance to notify at least one partner [80]. Moreover, street-based outreach to partners in that study yielded additional opportunities to provide HIV risk reduction and distribute HIV prevention materials such as condoms and bleach. In Indonesia, a high proportion of PWID practice safe injection [2, 81]. Yet, fewer PWID have accessed opioid agonist treatment or HIV testing, and ART coverage among HIV-positive PWID (27%) also is low [81]. In this context, partner notification could, in principle, supplement existing efforts to achieve more widespread testing of those at risk, the first of the UNAIDS 95-95-95 goals for 2030, and provide earlier treatment to persons already infected [68].

This study is, to our knowledge, the first to explore the acceptability of HIV partner services in prisons and provides important new insights as to the potential risks and benefits of partner notification from the perspective of incarcerated PLHIV, a group that is vastly underrepresented in the partner services literature [82]. Based on a relatively large and representative sample of PLHIV, we provide detailed estimates for the number and characteristics of potentially notifiable partners, which may be useful for establishing a contact tracing period and allocating resources for future intervention work. Guided by an explicit theory, our analysis of interpersonal and psychosocial factors influencing disclosure provides a roadmap for developing disclosure decision aids and other tools to support voluntary and informed decision-making in these settings. Set in a country where many PLHIV are diagnosed in prison, our findings may usefully guide work in other LMICs with HIV epidemics likewise concentrated in prisons.

The current study is subject to several limitations. Presented with a hypothetical scenario, participants may have responded in a manner that they felt was more socially acceptable, and previous studies suggest that hypothetical willingness may overestimate behavioral intentions [83]. Participants were not asked which partner notification method they would prefer, nor were they asked about their sex/drug-injecting partners within prison who also may benefit from HIV testing. Our cross-sectional design does not allow us to infer the direction of associations and absence of an association could be due to insufficient power. Most study measures were developed for community populations and may be less reliable or reflective of the concerns of people in prisons. Participants in this study were asked to generalize about their interpersonal relationships, yet disclosure decisions are likely made based on consideration of the specific circumstances of each individual relationship. Further studies are needed to understand which of these factors most influence individual decisions as to whether and how to notify partners. Finally, participants were recruited from just two male prisons and not randomly selected, which limits generalizability, including to female prisoners, who are a smaller share of Indonesia’s prison population (5.5%) [28] but have higher HIV prevalence (6.0%) [29].

## Conclusion

PLHIV in prison are often the last to receive internationally-recommended HIV prevention and treatment services, to the detriment of individual and public health. Assisted HIV partner services are demonstrably safe and effective in reaching people with undiagnosed infection and may prove even more potent when offered to PLHIV in prison as part of a comprehensive package of HIV prevention and treatment services. As this study shows, PLHIV in prison understand the importance of notifying their partners and are prepared to accept notification assistance from healthcare providers. Despite their present circumstances and the possibility that their partners may reject them, a majority of PLHIV in these prisons are committed to seeing that their partners receive information about HIV testing, treatment, and support. Provider-assisted approaches offer a simple and potentially effective way to notify these partners and could alleviate some of the difficulties and concerns that PLHIV have about notifying partners themselves. Given the recentness of their diagnosis, the high frequency of drug and sex risk behaviors before incarceration, and their expressed willingness to notify partners, PLHIV in prison are excellent candidates for partner services. Assisted partner notification for PLHIV in prison may provide a targeted and resource conserving approach to confront emerging challenges within the current HIV epidemic in Indonesia, including rapidly increasing new infections and barriers to HIV testing in women and a surge in drug-resistant HIV among PWID that is tied to prisons. While further studies are required to ascertain the actual risks and benefits of implementing partner services in prisons, this study shows that lack of acceptability is no longer an argument for withholding this vital assistance.
